# Water Hyacinth Cellulose for Polymer Composites and Bioplastics: A Review of Extraction, Formulation and Applications

**DOI:** 10.3390/polym18212797

**Published:** 2026-11-09

**Authors:** Oluwaseyi Matthew Abioye, Oluwaseun Temitope Faloye, Timothy Adesoye Adekanye, Matthew Folorunsho Amodu, Abiodun Afolabi Okunola, Oluwafemi E. Adeyeri, Laemthong Laokhongthavorn, Viroon Kamchoom

**Affiliations:** 1 Department of Agricultural and Biosystems Engineering, Landmark University, Omu-Aran PMB 1001, Nigeria; abioye.oluwaseyi@lmu.edu.ng (O.M.A.); okunola.abiodun@lmu.edu.ng (A.A.O.); 2 Department of Water Resources Management and Agrometeorology, Federal University, Oye-Ekiti PMB 373, Nigeria; oluwaseun.faloye@fuoye.edu.ng; 3 Department of Agricultural and Biosystems Engineering, Federal University of Technology and Environmental Sciences, Iyin Ekiti PMB 001, Nigeria; timothy.adekanye@futes.edu.ng; 4 ARC Centre of Excellence for the Weather of the 21st Century, Fenner School of Environment and Society, The Australian National University, Australian Capital Territory, Canberra 2600, Australia; 5 Excellent Centre for Green and Sustainable Infrastructure, School of Engineering, King Mongkut’s Institute of Technology Ladkrabang, Bangkok 10520, Thailand

**Keywords:** circular bioeconomy, water hyacinth, polymer composites, bioplastics, cellulose nanocrystals, value-chain framework, invasive species valorization

## Abstract

Petroleum-based plastics generate over 400 million tonnes of waste annually, posing severe ecological threats to terrestrial and aquatic environments. Concurrently, water hyacinth (*Eichhornia crassipes*, Mart.) infests over 50 countries, disrupting waterways, fisheries, and riparian economies. This review proposes that both challenges can be addressed within a single material strategy: valorizing water hyacinth biomass into cellulose-derived bioplastics, nanocellulose composites, and functional hydrogels. To organize the evidence, the review uses a conceptual Harvest–Extract–Formulate–Deploy (HEFD) scheme that follows the value chain from field harvest through cellulose extraction, composite formulation and crosslinking to end use. The scheme is a structuring device and not a validated model. The review draws on the peer-reviewed literature published between 2018 and 2026 and examines composition, mechanical and thermal performance, synthesis routes, and applications in packaging, agriculture and biomedicine. Water hyacinth is not a plastic in itself but a variable lignocellulosic raw material, and the properties of materials made from it depend mainly on the matrix polymer, filler content, and the interface between the two. Reported tensile strengths are modest and elongation at break is far below that of low-density polyethylene (LDPE), so equivalence with LDPE has not been shown. Mass loss in soil-burial tests ranges from a few percent to near-complete disintegration depending on the formulation, and few studies measure mineralization. Feedstock may be cheap, or even paid for, where removal is publicly funded, but no peer-reviewed techno-economic study has confirmed this advantage. A decentralized small-scale production model is proposed for tropical smallholder settings, and clear gaps remain in life-cycle assessment, standardization, and regulation.

## 1. Introduction

A long-standing and ongoing global crisis is intensifying owing to our relationship with plastics. The world’s industrial economy has adopted and integrated plastic materials for their characteristics, such as lower material costs, low manufacturing weights, high inertness to other materials, and long product life expectancy. Unfortunately for society, the long-lasting ability of plastics has transitioned from a technological advancement to an environmental calamity. Currently, the annual production of plastic is more than 400 million tons [1], but only about 9% of plastic waste was recycled worldwide in 2019 [2,3]; an earlier global assessment found a similar share (9%) for all plastic made up to 2015 [4]. Most of the rest has been disposed of through landfills or incineration, or has been released into our ecosystems, where it will degrade over hundreds of years into microplastics that are currently found in polar ice, in ocean sediments and in soils [5,6]. Reports of microplastics keep accumulating, and concern about their effects on ecosystems and human health is growing. Chemical recycling routes, such as the alkaline hydrolysis of PET, are being developed to raise the recycled share, but their net benefit depends on how the whole system is designed [7]. The scientific and policy communities increasingly argue that, although these improvements to recycling and waste management infrastructure are necessary, they are still insufficient. What is required is a fundamental change in the materials that we use, a change to a sustainable materials future, and a change to materials that are not non-degradable, fossil fuel-based plastics, but rather materials that are bio-based, biodegradable, and, if possible, recyclable or circular [8,9]. The best near-term hope for such a change is the development and use of bioplastics, plastics that are produced from biological sources. There is an increasing body of evidence that indicates that cellulose-based, starch-based, PLA-based, and PHA-based bioplastics can approach the performance of conventional plastics in some applications and, depending on how the system is defined, can lower greenhouse gas emissions and offer better end-of-life options [10,11]. The problem for the emerging bioplastics industry, however, is that competition for terrestrial biomass for use as a feedstock for these plastics and competition between bioplastic feedstock production and food crop cultivation—the so-called food–materials land-use conflict—limits the scalability of starch and sugar-based bioplastic supply chains [12].

It is due to this very limitation that *Eichhornia crassipes*, commonly referred to as water hyacinth, enters the scene, not as a traditional feedstock, but as a genuinely disruptive one. Water hyacinth is widely regarded as one of the world’s most prolific aquatic weeds [13]. Originally introduced to the Amazon basin, it is now distributed across over 50 countries in tropical and subtropical Africa, Asia, and Australasia, forming large floating mats that obstruct waterways, crowd out native biodiversity, decrease dissolved oxygen, limit fishing opportunities, and harm hydroelectric activity [14,15]. Manual and chemical removal programs cost governments and water authorities large sums each year and often provide only short-term relief, because the plant can double its biomass within a few weeks under good conditions [16]. Therefore, this invasive species is also a plentiful and, in many places, low-cost source of biomass.

Water hyacinth is a candidate raw material for cellulose-based materials because of its chemical composition, although that composition is variable. Reported cellulose contents range from approximately 20% to 68% of dry matter and hemicellulose from approximately 11% to 33%, while lignin is usually below approximately 10% in whole-plant and fiber analyses. Woody feedstocks and sugarcane bagasse generally contain more lignin, which makes cellulose extraction more demanding [17]. Lower lignin should allow milder pretreatment and shorter processing, which could reduce reagent use and cost; however, to our knowledge, no like-for-like comparison with other feedstocks has been published. Because the plant grows on water, harvesting it from infested waters requires no agricultural land, irrigation, or fertilizer, and the plant takes up nitrogen and phosphorus from the water, so harvesting also removes some nutrients. The same uptake means that plants from polluted water can accumulate heavy metals, which matters for materials that will touch food, soil, or skin (Section 2.2) [18].

Although this dual-benefit proposition may seem intuitive, the current body of information on water hyacinth as a bioplastic feedstock remains fragmented across materials science, environmental engineering, agronomy, and polymer chemistry, obscuring the full picture for stakeholders. Most existing reviews treat water hyacinth management and bioplastic development as separate topics. Water hyacinth biorefinery and policy studies already describe value chains for biogas, bioethanol, fertilizer, and cellulose [17,19,20], and frameworks that couple life-cycle assessment with techno-economic analysis exist for cellulose nanomaterials [21]. To our knowledge, however, no earlier review has organized the water hyacinth literature around the link between harvest-stage decisions (origin, contamination, and drying) and the polymer-level properties of the final material. This review uses the Harvest–Extract–Formulate–Deploy (HEFD) scheme for that purpose. HEFD is a conceptual organizing framework. It is not a validated optimization model or a decision-support tool; it has not been tested against case-study data, but it adds a way of grouping existing evidence and showing where the choices made in one layer affect the next. Specifically, this review aims to: (i) critically characterize the chemical, mechanical, and thermal properties of water hyacinth biomass and its derived materials; (ii) evaluate synthesis and formulation strategies, including extraction, composite design, and crosslinking; (iii) assess multi-sector application evidence across packaging, agriculture, and biomedical sectors; (iv) assess what is known about environmental and socio-economic performance; and (v) identify standardization gaps and propose a research agenda for commercially scalable, regulation-compliant water hyacinth bioplastic value chains.

###  Literature Search and Selection 

**Figure 1:**
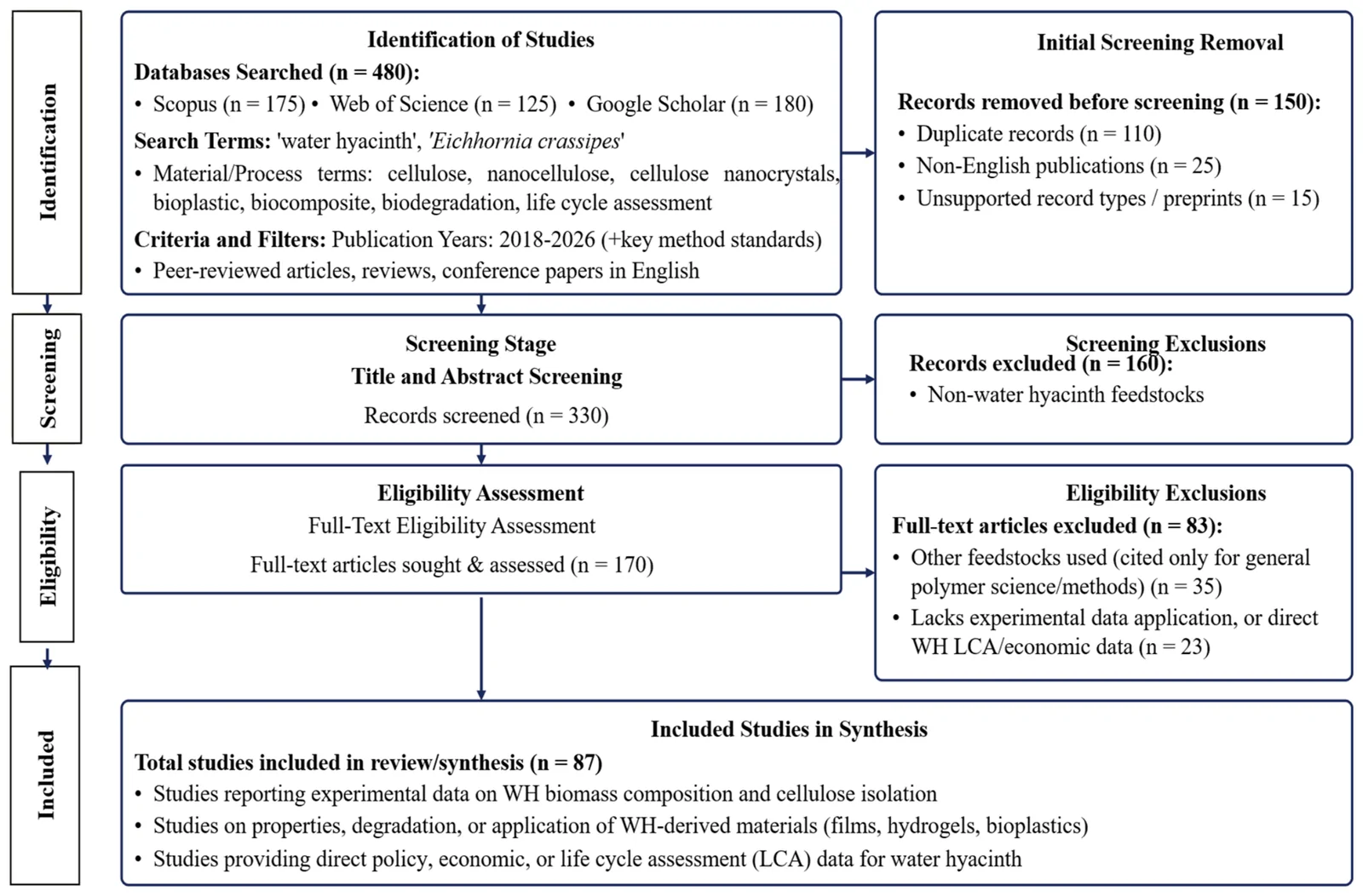
PRISMA flow diagram of the literature search and study selection.

The literature was searched in Scopus, Web of Science, and Google Scholar. Search strings combined water hyacinth terms (“water hyacinth,” “*Eichhornia crassipes*,” “*Pontederia crassipes*”) with material and process terms such as cellulose, nanocellulose, cellulose nanocrystals, carboxymethyl cellulose, bioplastic, biocomposite, film, hydrogel, mulch, biodegradation, and life cycle assessment. Records published between 2018 and 2026 were considered, and a few older papers were retained where they were the primary source of a method, a number, or a standard. Peer-reviewed articles, reviews, and conference papers in English were retained. A study was used as evidence about water hyacinth if it reported experimental data on the composition of the biomass, the isolation of cellulose, or the properties, degradation, or application of water hyacinth-derived materials, or if it gave policy, economic, or life-cycle information directly about water hyacinth. Titles and abstracts were screened first, and full texts were read for the studies that remained. Work on cellulose-based materials from other feedstocks was not treated as evidence about water hyacinth. It is cited only for general polymer science, standards, or methods, and the text says where this is the case. Studies were not scored with a formal quality tool and no meta-analysis was attempted because materials, test methods, and reporting differed too much between studies. Figure 1 shows the PRISMA flow chart for the literature search.

## 2. Chemical and Physical Properties of Water Hyacinth Biomass

### 2.1. Compositional Analysis and the Low-Lignin Advantage

Water hyacinth is an herbaceous aquatic macrophyte whose vegetative organs, petioles, leaf lamina, and stolons are predominantly composed of a lignocellulosic matrix. However, published analyses do not agree on a single composition. Cellulose has been reported to be present in approximately 20% to 68% of dry matter, hemicellulose from approximately 11% to 33%, and lignin from approximately 7% to 10% in whole-plant and fiber samples, with a much higher lignin value (24%) in one isolated cellulose fraction. The rest is protein, ash, and extractives [17]. The differences come from the plant part analyzed, the analytical method, the growth site, and the season; therefore, the values should not be read as a consensus. The cellulose fraction is not exceptional, and many agricultural residues carry a comparable or greater amount. Lower lignin in most analyses may nevertheless make pretreatment easier than for woody feedstocks. However, the main impediment in the extraction of cellulose from the biomass of plants is the presence of lignin, which coats the cellulose fibers with a protective shell of aromatic compounds that can only be destroyed by vigorous chemical or thermal treatment of the biomass. The most common methods of pretreating biomass are high-temperature alkaline treatment, acid treatment, and bleaching with oxidizing agents. All these methods result in the production of effluent, the use of considerable amounts of energy, and the degradation of some of the target cellulose material. In biomass with comparatively low lignin, such as water hyacinth (about 7–10% in the whole-plant and fiber analyses in Table 1, against roughly 15–25% typical of hardwoods), milder conditions may be sufficient. Reported cellulose purities after pretreatment vary widely. An optimized organosolv–alkaline process gave 92.98 wt% cellulose purity and raised the crystallinity index from 37.92% to 73.52% [22], whereas a bleaching–alkalination isolate contained 53.45% cellulose and 24.43% lignin [23]. Dilute alkaline pretreatment has been studied mainly to release xylose from the biomass, and it raised the crystallinity index of the biomass from about 26% to about 52% [24]. Milder conditions could cut reagent and energy use and the volume of effluent, but no study we found has quantified this for water hyacinth. Aside from the macromolecular makeup of the plant, the unusual ability of *Eichhornia crassipes*, also known as water hyacinth, to uptake nutrients from its aquatic surroundings offers an interesting level of compositional diversity. Plants from nutrient-rich (eutrophic) waters may be expected to contain more protein and relatively less structural carbohydrate than plants from nutrient-poor waters, but systematic comparisons of cellulose yield and quality between sites are scarce, and this is probably one reason for the spread of values. Lignin and other residual fractions are being explored as functional fillers in biopolymer matrices [25], and process-design studies discuss how such co-products can be integrated into biorefineries [26]. The systematic upgrading of the hemicellulose and lignin co-fractions, which are discarded as process waste in many designs, may be an opportunity that has received little attention for water hyacinth. Table 1 shows the percentage composition of the biomass and fiber present in water hyacinth.

### 2.2. Biomass Origin, Contamination and Their Consequences

Water hyacinth takes up dissolved nutrients and metals; therefore, the biomass reflects the water in which it grows. In a study of *Eichhornia crassipes* in wastewater, root-to-shoot translocation factors above 1 were found for several metals (Zn 3.62 in freshwater; Cd 2.34 in urban wastewater; Cr 1.61 and Pb 1.29 in industrial wastewater), and bioconcentration factors were higher in roots than in shoots [18]. Metal load can also affect what is done with the biomass: in one study that combined phytoremediation with bioethanol production, chromium in the plants was linked to a 25% fall in ethanol output [31]. Reporting of contaminant levels in water hyacinth used for agricultural inputs has been inconsistent [32].

For materials, this matters in three ways. First, metals and organic contaminants that survive isolation could end up in a film or hydrogel, which would be unacceptable for food-contact, agricultural soil, or biomedical uses. Second, contaminated biomass may require additional washing or acid treatment, which adds chemicals, water, and wastewater to the process, and residual alkali, bleaching agents, or solvents from purification can remain in the cellulose. Third, harvesting from polluted sites exposes workers to pathogens and contaminants. Most of the studies do not report the metal content of the feedstock or of the isolated cellulose; therefore, the suitability of water hyacinth cellulose for packaging, agricultural, or biomedical use cannot be judged from the present literature. In practice, biomass from sites with known contamination should be screened, and, where the limits for the intended use are exceeded, it should be directed to uses without food or skin contact. Studies should also report the origin of the biomass, the season of harvest, and the metal content alongside the material properties.

### 2.3. Mechanical Properties of Water Hyacinth-Derived Materials

The mechanical properties of bioplastics are not an intrinsic property of the material but rather an attribute that is dependent on the formulation of the bioplastics, including cellulose content, the type of matrix polymer used, plasticizer content, and processing conditions. This is an important consideration when discussing the literature, which spans a wide range of conditions. The studies we found do not point to a single optimum filler content. Tensile strength peaked at 12% cellulose (2.55 MPa) in Kepok banana peel starch filled with water hyacinth cellulose, while mass loss was greatest at 4% cellulose [33]. Corn starch films with 0–10% water hyacinth CNC showed fluctuating tensile strength and elongation, with the greatest mass reduction at 6% CNC [34]. Water hyacinth cellulose powder has also been used as a reinforcing agent in starch bioplastic [35]. Beyond an optimum loading, aggregation lowers performance [36]. Elongation at break of water hyacinth composites is generally far below that of LDPE, which typically stretches by several hundred percent, so these composites are poorly suited to uses that need high ductility, such as stretch wrapping or heavy-duty bags. Reported tensile strengths for starch-based water hyacinth films are also low, and equivalence with LDPE has not been demonstrated. In thermoplastic starch, nanocellulose forms hydrogen bonds with the starch and the plasticiser, and the type and amount of plasticiser set how mobile the starch chains are. In one starch–CNC system (not water hyacinth), adding CNC raised tensile strength from 3.7 to 7.1 MPa with 40% glycerol and from 11.1 to 27.1 MPa with 30% sorbitol [37]. PLA is hydrophobic and cellulose is hydrophilic, so the two mix poorly. In cellulose/PLA blends, cellulose pull-out and a marked loss of impact strength and elongation were seen, and a cellulose-graft-PLA compatibiliser largely restored the properties [38]. The cellulose–matrix interface is a major factor in mechanical performance. Hydrogen-bonding patterns govern the strength of CNC, aggregation beyond an optimum loading lowers performance, and a rigid CNC network raises the storage modulus and the glass transition temperature [36]. For rod-like CNC, the aspect ratio determines how readily a percolating network forms, and simple series–parallel models fit low-aspect-ratio CNC poorly [39]. In starch-based bioplastics filled with acid-treated cellulose, scanning electron microscopy (SEM) has shown that a uniform fracture surface is obtained if the cellulose fibers are well dispersed and the matrices are sufficiently plasticized, while agglomerations or poor interfacial bonding may cause debonding at the interface, resulting in premature failure [40].

Water hyacinth cellulose is not a single compound. It is a mixture in which cellulose, hemicellulose, residual lignin, and ash occur in proportions that depend on the plant part, site, and isolation route, and reported crystallinity indices run from approximately 26% in untreated biomass to approximately 74% after organosolv–alkaline treatment [22,24]. Crystalline cellulose domains are stiff and relatively impermeable; therefore, a higher crystallinity index generally corresponds to a stiffer filler and lower water uptake, whereas amorphous cellulose and hemicellulose are more hydrophilic and take up water more readily. Residual lignin and hemicellulose also change the surface chemistry of the filler and, therefore, its adhesion to the matrix.

The matrix adds its own interactions. In starch, glycerol and cellulose compete for hydrogen bonds with starch chains; the plasticizer lowers the glass transition temperature and increases chain mobility, and ageing leads to retrogradation and recrystallization [37]. Polyvinyl alcohol is a semi-crystalline polymer that can hydrogen bond with the hydroxyl groups of cellulose. The CMC is anionic, and its degree of substitution controls its solubility. PLA is a hydrophobic polyester that weakly interacts with cellulose unless a compatibilizer, silane, or surface treatment is used [38]. Crosslinkers add another layer: citric acid forms ester links with hydroxyl groups, borax forms reversible complexes with cis-diols, and radiation can create covalent bonds between chains. Each tightens the network and reduces water uptake; however, each can also make the material more brittle. These relationships follow general polymer science. Most of the water hyacinth studies cited here characterize the final material by tensile tests and mass loss, rather than by methods that probe these interactions directly, such as infrared spectroscopy, thermal analysis, dynamic mechanical analysis, or X-ray diffraction. Closing this gap would make the property data easier to compare and explain.

### 2.4. Thermal and Degradation Characteristics

Five terms are kept apart in this review. Mass loss is the decrease in the weight of a sample after burial, immersion, or composting, regardless of the cause. Fragmentation is the physical breaking of a sample into smaller pieces without a change in the polymer chemistry. Dissolution is the loss of material into water, which is important for water-soluble matrices such as CMC and PVA. Microbial degradation is the enzymatic breakdown of the polymer by microorganisms, which is shown, for example, by a fall in molecular weight or by respiration. Mineralization is the conversion of polymer carbon to CO_2_ (or to CH_4_ and CO_2_ under anaerobic conditions), and the term is used here only when biodegradation is quantified from gas evolution or oxygen demand, as in ISO 14855 and in anaerobic digestion assays. Where a study reports only weight loss or visual change, the text says so and does not call the result biodegradation.

Thermal stability matters for both processing and end use. Thermogravimetric analysis (TGA) of lignocellulosic materials typically shows moisture loss at low temperature, then degradation of hemicellulose, then of cellulose, and finally oxidation of the char; the temperatures depend on composition, crystallinity and matrix. Thermal data specific to water hyacinth composites are sparse in the studies we reviewed, and comparisons with LDPE or polypropylene should be made only under identical test conditions. Biodegradation, the capacity of a material to be mineralised by microorganisms under defined environmental conditions, is the property that most clearly differentiates water hyacinth bioplastics from petroleum-based counterparts. Reported degradation results for water hyacinth materials cannot be compared directly, because the tests differ. A corn starch film filled with water hyacinth CNC showed its greatest mass reduction at 6% CNC on day 10 [34]. Films of Kepok banana peel starch with water hyacinth cellulose lost 63.18% of their mass in the best case, at 4% cellulose [33]. A water hyacinth CMC–tapioca starch film buried at 10 cm lost about 10% of its weight in 24 h and was reported as fully decomposed after 120–144 h, depending on glycerol content [41]. These are mass-loss tests. They show disintegration under the test conditions and do not show mineralisation. ISO 14855-1 measures the CO_2_ evolved under controlled composting conditions [42], whereas ASTM D5988 measures aerobic biodegradation of plastics in soil from evolved CO_2_ relative to a reference [43]. The two standards differ in the test environment (compost or soil) and in the quantity measured, and neither is a simple soil-burial mass-loss test, so they are not interchangeable. Mass loss in a burial test should not be cited as compliance with either of them. An important distinction, seldom made clear, is that between disintegration (physical fragmentation, which can be rapid but does not show conversion to inorganic end-products) and mineralisation (conversion to CO_2_ or CH_4_ and water). Table 2 outlines the primary measurement methods, quantitative metrics, and representative studies evaluating the biodegradation of water hyacinth biomass and derived bioplastics under anaerobic digestion conditions.

## 3. The HEFD Framework: From Field Harvest to Deployable Material

The Harvest–Extract–Formulate–Deploy (HEFD) valorization model presented in this review represents the water hyacinth bioplastic value chain in four consecutive yet closely interrelated decision layers. In contrast to a purely technical process description, the HEFD framework is multidimensional: each layer encompasses technical parameters alongside economic, environmental, and logistical implications that govern real implementation. The key stages, process parameters, and operational strategies of the Harvest–Extract–Formulate–Deploy (HEFD) valorization framework for the production of water hyacinth bioplastics are detailed in Table 3.

### 3.1. Harvest Layer: Managing the Feedstock Paradox

The paradox at the harvest layer is that the very characteristics responsible for water hyacinth’s ecological destructiveness, rapid biomass accumulation, year-round growth in tropical environments, and proliferation in nutrient-rich polluted waters, simultaneously make it a valuable industrial feedstock. Dense infestations can produce large amounts of fresh biomass with almost no cultivation effort, although yields vary with climate, nutrient load and season [55,56]. Where agencies or water utilities are obliged to clear waterways, they may pay for removal, so the biomass could reach a processor at low or even negative cost. We found no published techno-economic study that tests this for water hyacinth bioplastics, and the cost of collection, transport and drying could cancel it. It should be treated as a hypothesis.

The logistics of harvesting water hyacinth are not trivial. Manual harvesting, which is the dominant method in smallholder systems in sub-Saharan Africa and South-East Asia, is labour-intensive and exposes workers to occupational hazards associated with waterborne pathogens and bioaccumulation of heavy metals and other non-biodegradable chemicals in plant tissues due to water body pollution. Mechanized harvesting equipment is effective for large, navigable water bodies but is costly and requires supporting infrastructure for receiving and dewatering harvested water hyacinth. Freshwater hyacinth contains roughly 90–95% water by mass and can carry waterborne pathogens, so prompt dewatering and drying to a moisture content below about 12% is needed before any chemical treatment. Where the plants come from polluted water, they may also carry metals and other contaminants, so the origin of the biomass matters as much as the logistics (Section 2.2). The least expensive method, especially in tropical regions, is represented by the sun-drying method, although it is the slowest and depends on weather conditions. On the other hand, the forced air or drum method is more controllable, although energy costs are involved. The energy requirement in the harvest stage, although not considered in optimization calculations starting with dry biomass, is an important factor in the energy balance [17].

### 3.2. Extract Layer: Cellulose Isolation Strategies

Cellulose is extracted from the water hyacinth through a series of chemical, mechanical, and biological treatments. The most common route is alkaline treatment followed by bleaching with hypochlorite or chlorite, as used for other lignocellulosic materials. The outcome depends strongly on the conditions. A bleaching–alkalination procedure produced a fibre with 53.45% cellulose, 24.43% lignin and 22.12% hemicellulose [23], while an optimized organosolv–alkaline process reached 92.98 wt% cellulose purity [22]. Preparation of cellulose nanofibrils from water hyacinth gave an extraction yield of 19.38% [48]. The alkaline–bleaching route is well understood and can be run at community scale with little equipment, but it consumes chemicals and produces effluent that has to be treated.

For processes requiring high purity, advanced pretreatment techniques are being increasingly adopted. Organosolv pretreatment, using organic solvents like ethanol or acetone, along with water and a catalyst, simultaneously solubilizes hemicellulose and lignin in one step and can produce a lignin co-product. An organosolv–alkaline route for water hyacinth has been optimized by response surface methodology and accompanied by a preliminary life-cycle and techno-economic assessment [22]. Deep eutectic solvents, which consist of hydrogen bond donors and acceptors, have been used to delignify lignocellulosic biomass, including under microwave heating [57], and are discussed as lower-toxicity, recyclable alternatives to conventional organic solvents, although cost and solvent recovery limit scale-up [49]. We did not find a study that applied them to water hyacinth in the sources reviewed. Enzymatic delignification is more selective in principle, but it is generally regarded as too costly for industrial cellulose production.

**Figure 2:**
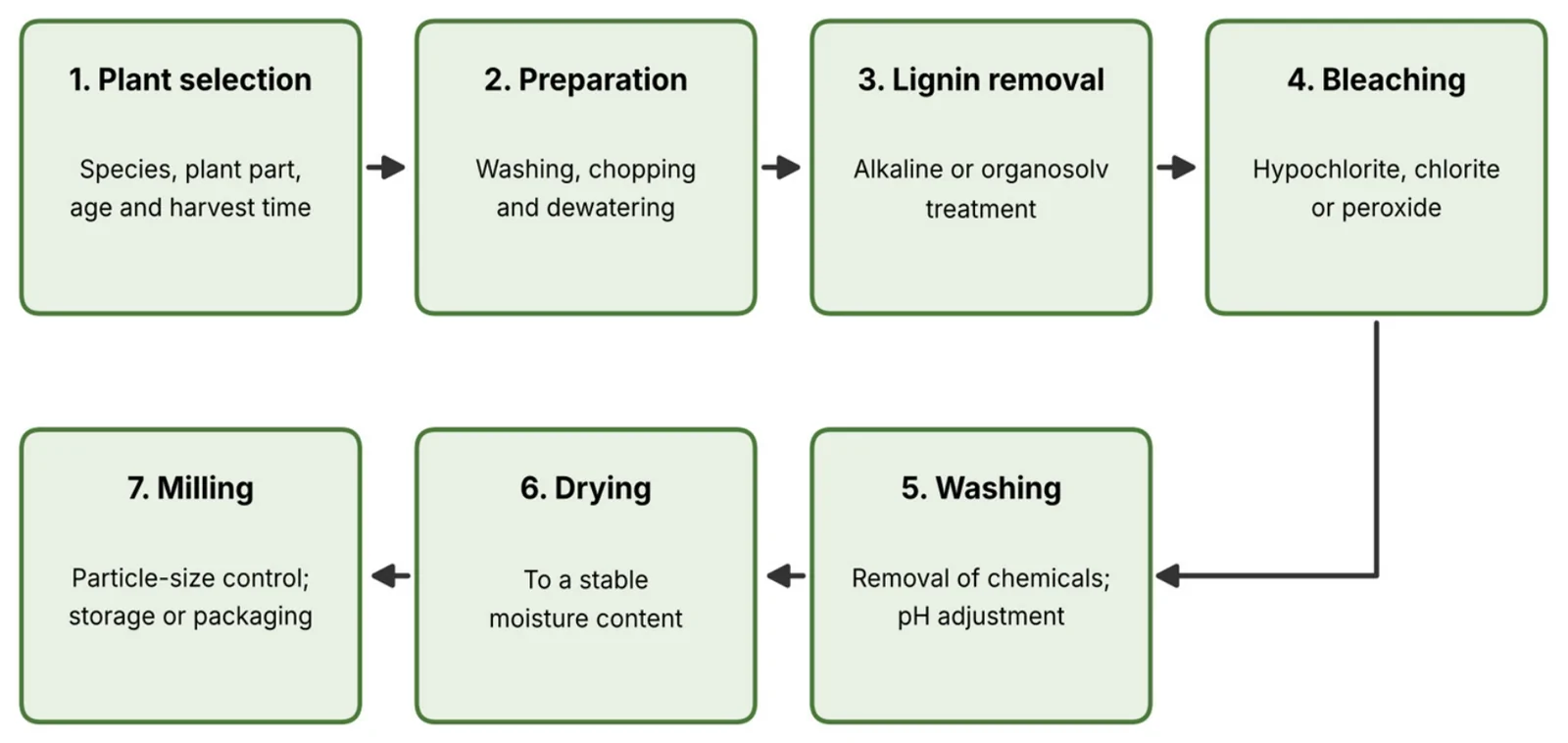
Stages of cellulose extraction from water hyacinth (extract layer of the HEFD scheme): selection of plants, preparation of the material, lignin removal, bleaching, washing and pH adjustment, drying and milling.

Further processing of cellulose into microcrystalline cellulose (MCC) or cellulose nanocrystals (CNC) is possible using acid hydrolysis. Acid hydrolysis removes the amorphous regions and leaves rod-like, highly crystalline particles whose reinforcing effect depends on their aspect ratio and on how well they are dispersed [36,39]. Water hyacinth CNC has been incorporated into polyvinyl alcohol/gelatin films, and the packaging and degradability properties of those films were reported [58]. For CMC production, extracted cellulose is carboxymethylated through reaction with sodium monochloroacetate under alkaline conditions, yielding a water-soluble derivative whose degree of substitution (DS) controls solubility and film behaviour. CMC from water hyacinth has been blended with tapioca starch and glycerol to make films [41], and starch-based CMC films have also been made from rice husk and straw [59]. Figure 2 summarizes the cellulose extraction process from water hyacinth.

### 3.3. Formulate Layer: Composite Design and Crosslinking

Bioplastic composites based on cellulose derived from water hyacinth plants include the choice of polymer matrix, cellulose content and shape, and choice of processing method. A widely used polymer matrix is gelatinized starch from potato, cassava or corn, plasticized with glycerol or sorbitol. This bioplastic is processed to produce flexible and transparent films using solvent casting. Citric acid is used to improve the barrier properties and strength of bioplastics. Citric acid acts both as plasticizer and a crosslinking agent, forming ester linkages with hydroxyl groups on the starch molecules, thus reducing the sensitivity to water, which is the major limitation of bioplastics based on starch [60,61].

Crosslinking is an important parameter, often suboptimized. Gamma irradiation can form covalent crosslinks between carboxymethyl cellulose chains, which in general raises rigidity and reduces water uptake, but we did not find a water hyacinth study that reports the dose–property relationship, so its value for this material remains to be shown. Borax, or sodium tetraborate, is a crosslinking agent for the reversible formation of physical crosslinks with the cis-diol groups of cellulose and starch, making it a potential agent for hydrogel formation. Mercerization, or increasing the surface density of hydroxyl groups through dilute alkali, or even silane coupling to improve compatibility with hydrophobic matrices such as PLA, is crucial for hybrid composites owing to the hydrophilic nature of cellulose itself [62].

## 4. Multi-Sector Applications of Water Hyacinth Bioplastics

### 4.1. Packaging Industry

Packaging is the largest end use of conventional plastics, which makes it the most obvious target market for water hyacinth materials. The technical requirements have been well defined, including tensile strength, water vapour transmission rate (WVTR), grease and oil resistance, transparency, sealability, and food safety standards. To our knowledge, no single study has evaluated a water hyacinth material against all of these requirements, and the results so far are mixed.

Tensile strengths reported for starch-based water hyacinth films are low. The highest value in the banana peel starch study was 2.55 MPa [33], and the strength of corn starch–CNC films varied with the CNC content [34]. Films with this performance are, at best, candidates for dry or light-duty uses, and any claim of suitability for food packaging needs tensile, barrier and migration data for the specific application. Films based on hydrophilic cellulose and starch are generally more permeable to water vapour than LDPE, which makes them less suitable for moisture-sensitive foods. Two common remedies are adding nanocellulose and using a multilayer structure, with the bioplastic as the outer layer and a moisture barrier as the inner layer [63]. In thermoplastic starch films (not water hyacinth CNC), water vapour permeability fell steeply with CNC contents up to 20 wt% and then stayed almost constant up to 50 wt% [37]. From a circular-economy perspective, the end-of-life benefit of compostable packaging depends on certification. EN 13432 sets requirements for packaging recoverable through composting and biodegradation [50], and ASTM D6400 is the corresponding North American specification for plastics designed to be composted [64]. A material does not meet these standards because it loses mass in a soil-burial test; it has to pass the full test scheme. We did not find a water hyacinth packaging material that has been certified to either standard. Where certified compostable packaging does exist, it also needs industrial composting facilities and a collection route, which are not available everywhere. CMC is a permitted food additive (E466) in the European Union and is generally recognized as safe (GRAS) in the United States, but approval of an additive grade does not extend to packaging made from water hyacinth CMC. Food-contact plastics are regulated separately: in the EU by Regulation (EU) No 10/2011, which sets migration limits [51], and in the United States by FDA rules (21 CFR). Residues from processing, such as bleaching agents, alkalis and plasticisers, and any contaminants taken up from the water must be measured in migration tests on the finished article [11]. Table 4 summarizes the composition, processing and reported performance of water hyacinth-derived materials as stated in the source studies. The rows come from different test methods and are not directly comparable.

### 4.2. Agricultural Applications

The circular logic of water hyacinth materials is attractive for agriculture, but their practical performance is still largely unproven. The same plant that impairs irrigation infrastructure and reduces fishing yields can, when valorized, produce biodegradable mulch films, seedling pots, controlled-release fertilizer carriers, and soil-conditioning hydrogels that could support crop production and leave less persistent polymer residue in soil than conventional films, provided that degradation is demonstrated under field conditions, a problem of mounting concern as the literature on soil microplastic contamination from conventional agricultural films continues to expand [67,68].

In agriculture, water hyacinth has so far been used mainly as compost, mulch and a soil amendment [32], and empirical work from rural India describes turning the plant into agricultural inputs [69]. We did not find a field trial of a water hyacinth cellulose–starch mulch film compared with LDPE for weed control, soil temperature and moisture, so degradation rates in the field and equivalent agronomic performance remain to be shown. A biodegradable mulch film left in the soil would also need to meet EN 17033 [52]. If a film degraded fully, the remaining organic matter could add to soil carbon, but we found no measurement of this for water hyacinth films.

Cellulose-based superabsorbent hydrogels are reviewed as sustainable alternatives to synthetic polyacrylate hydrogels for water retention and nutrient delivery [70]. We did not find a study of water hyacinth-derived hydrogels applied in soil, so a water-retention benefit from this feedstock has not been shown. Such hydrogels could be a means of simultaneously delivering slow-release fertilizers while providing synchronized nutrients and water to the soil in order to increase fertilizer use efficiency, as well as to reduce the potential for leaching into water bodies. Reducing nutrient leaching matters because nutrient enrichment of water bodies is widely recognized as a driver of water hyacinth growth [16].

### 4.3. Biomedical and Emerging Applications

This subsection describes prospects, not demonstrated applications: we found no experimental biomedical data for water hyacinth materials. Bioplastics and cellulose-based nanomaterials made from the waste of the water hyacinth plant (*Eichhornia crassipes*) have the potential to provide innovations for biomedical and technical industries. Cellulose nanomaterials in general attract interest for biomedical use because they are biodegradable and can reinforce polymers at low CNC content. Preliminary biocompatibility evidence for cellulose nanocrystals from various sources [71,72] suggests potential applicability in wound healing and drug delivery. To our knowledge, no experimental validation of water hyacinth-derived CNC in biological systems has been published. Any biomedical use would also require control of metal and microbial contamination of the feedstock and biological evaluation under ISO 10993-1 [53].

**Figure 3:**
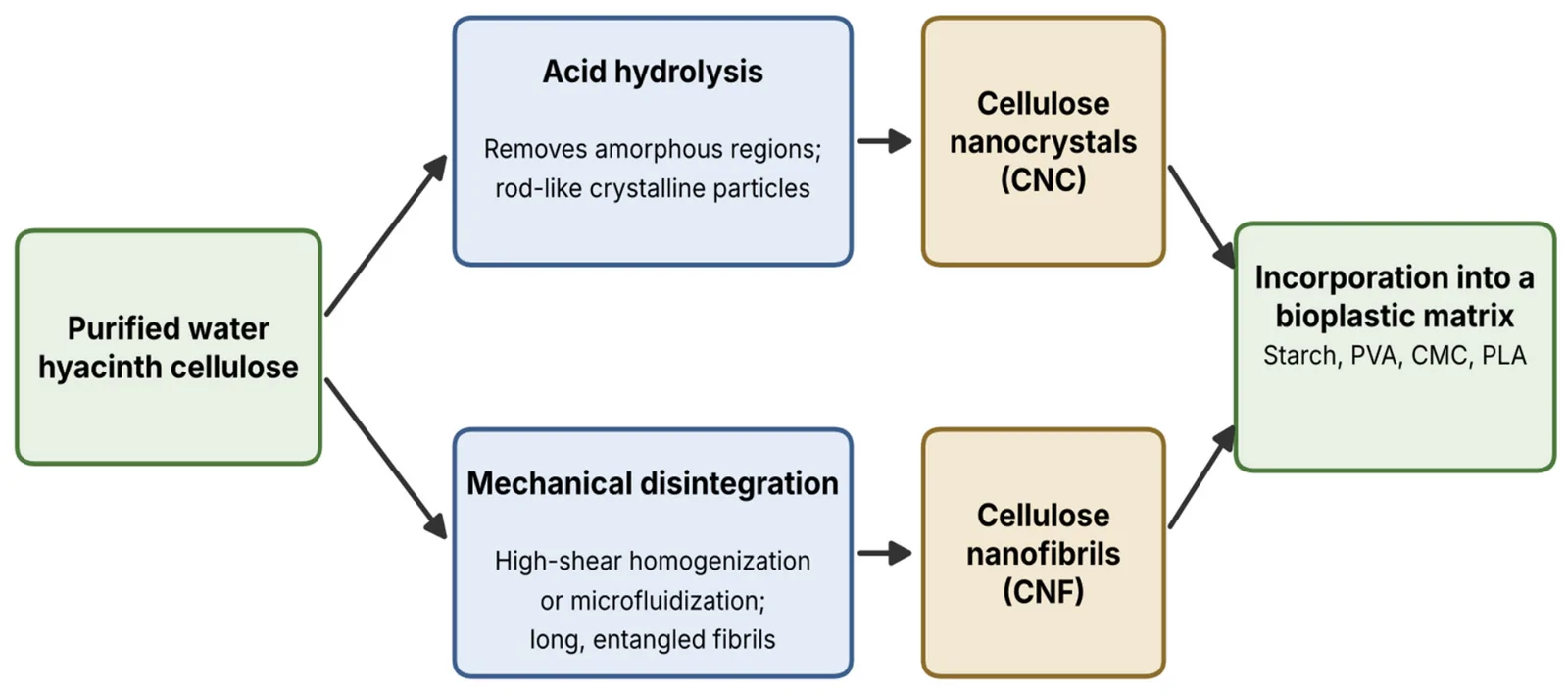
Qualitative overview of nanocellulose routes from water hyacinth and their expected effect on bioplastic properties.

For many years, Indonesia and the Philippines have used water hyacinth fibre to create woven textiles. The recent interest in developing natural fibres to make composite materials for constructing boards and making parts for vehicle interiors continues to build on this existing body of knowledge [62,73]. Water hyacinth fibre has been tested as a reinforcement in epoxy composites for lightweight applications [28]. Whether such composites can replace glass-fibre-reinforced polypropylene in non-structural automotive parts has not been shown. Figure 3 gives a qualitative overview of nanocellulose routes (acid hydrolysis to CNC and mechanical disintegration to CNF) and their expected effect on bioplastic properties.

### 4.4. Regulatory Routes by Application

Different applications fall under different rules, and compliance in one does not carry over to another. Plastic food-contact materials in the EU are regulated by Regulation (EU) No 10/2011, which sets migration limits [51]. Compostable packaging is assessed against EN 13432 [50] or ASTM D6400 [64], which cover biodegradability, disintegration and the effect on the composting process and compost quality. Biodegradable mulch films for agriculture are covered by EN 17033 [52], and fertilizing products by Regulation (EU) 2019/1009 [54]. Biological evaluation of medical devices follows ISO 10993-1 [53]. Regulation (EU) 2023/2055 amends Annex XVII of REACH as regards synthetic polymer microparticles intentionally added to products [74]; it is not a general framework for single-use food-contact materials and is not used as one here. Mass loss in a soil-burial test shows compliance with neither EN 13432 nor EN 17033.

## 5. Socioeconomic and Environmental Performance

### 5.1. Life-Cycle Environmental Assessment

While theoretically appealing, the ecological profile of water hyacinth bioplastics requires rigorous quantification to translate theory into practice. Life Cycle Assessment (LCA) provides the most thorough evaluation of the environmental impacts of product manufacture over their entire life cycle from raw material sourcing through end of life, expressed using commonly accepted units for: global warming potential (GWP), land use, water consumption and eutrophication potential.

We found no published, peer-reviewed LCA of a water hyacinth bioplastic that follows ISO 14040 and ISO 14044 [75,76], so this is a major gap. The points below are expectations drawn from related work, not results for a water hyacinth bioplastic. (i) Avoiding agricultural land and freshwater irrigation for feedstock production is a plausible advantage over crop-based feedstocks, but its size has not been quantified. (ii) Avoided disposal of the removed biomass could be credited in an LCA, but the credit depends on what would otherwise happen to the biomass (landfilling, incineration or composting) and on the system boundary, and it should be tested in a sensitivity analysis. (iii) Drying and chemical pretreatment are energy-intensive, so the net climate result is likely to be sensitive to the energy source [17,77]. LCA work on water hyacinth so far covers other products: a preliminary assessment of biorefinery routes to biomethane, bioethanol and fertilizer in the Lake Tana region [19] and a ReCiPe-based assessment of organosolv–alkaline cellulose extraction [22]. Neither covers a bioplastic, so no greenhouse-gas value for a water hyacinth bioplastic can be quoted and no comparison with LDPE or PLA is justified yet.

Environmental burdens have to be counted along the whole chain. Fresh biomass is mostly water, so drying is likely to be a major energy demand; sun drying avoids fuel but is slow and depends on the weather. Chemical use (alkali, bleach, acid or solvents), the wastewater they produce, solvent recovery in organosolv and deep eutectic solvent routes, transport of wet biomass, and extra washing if the biomass is contaminated all belong inside the system boundary. We did not find energy figures for cellulose isolation from water hyacinth. For nanocellulose from other feedstocks, the figure depends strongly on the route: in a gate-to-gate assessment of nanocrystalline cellulose from sugarcane trash, freeze-drying accounted for nearly all of the electricity use [78], and the energy needed for cellulose nanofibrils varies with the pretreatment chosen [79]. Such values cannot be transferred to water hyacinth.

Comparisons with conventional plastics need a defined functional unit, such as a given area of film that meets the same mechanical and barrier requirements, rather than a mass of material, and the same system boundary on both sides. Reviews of bioplastic LCAs find that many studies leave out the use phase and that fewer than half test their end-of-life assumptions in a sensitivity analysis [80].

To keep the strength of the evidence clear, the benefits discussed in this review fall into three groups. Demonstrated at laboratory scale: water hyacinth cellulose and CMC can be made into films and composites, and some of these lose mass in soil burial. Theoretically expected: lower land and water demand, milder pretreatment because of the lower lignin content, and avoided disposal impacts. Conditional on processing and system choices: any net greenhouse gas benefit, which depends on the energy source, the drying route, chemical recovery and the disposal route the biomass would otherwise have followed.

### 5.2. Circular-Economy Integration and Socio-Economic Dimensions

The circular-economy case for water hyacinth bioplastics rests on three reinforcing propositions: ecological restoration, resource efficiency, and socio-economic development. As far as ecological restoration is concerned, the argument for harvesting water hyacinth for bioplastics is quite simple: by removing water hyacinth from infested water bodies, the amount of biomass continues to decrease over time. This results in improved oxygen levels and light penetration into the water and higher aquatic biodiversity compared to non-infested water bodies. As a result, such a value chain could bring economic benefits to riparian communities that rely on fishing and farming, although this has not been measured for bioplastics [15,16].

While ecological restoration is the most obvious reason to produce bioplastics using water hyacinth, the economic side of producing bioplastics from water hyacinth is another very important reason for doing so in developing countries. Countries such as Indonesia, Kenya, India, Ethiopia, and Nigeria have all experienced significant infestations of water hyacinth, and small and medium-sized companies have been able to successfully transform water hyacinth into products such as handicrafts, biochar, animal feed additives, and compost. These provide examples of the technical and organizational frameworks required to develop a bioplastic feedstock supply chain [20,69]. In a decentralized processing model, community cooperatives would manage harvesting and primary drying, and regional hubs would process the cellulose, finish the products and distribute them through urban networks. Such a model could create jobs along the supply chain, and it might help water hyacinth products compete with imported packaging where tariffs or carbon taxes apply, but this has not been tested economically [56].

**Figure 4:**
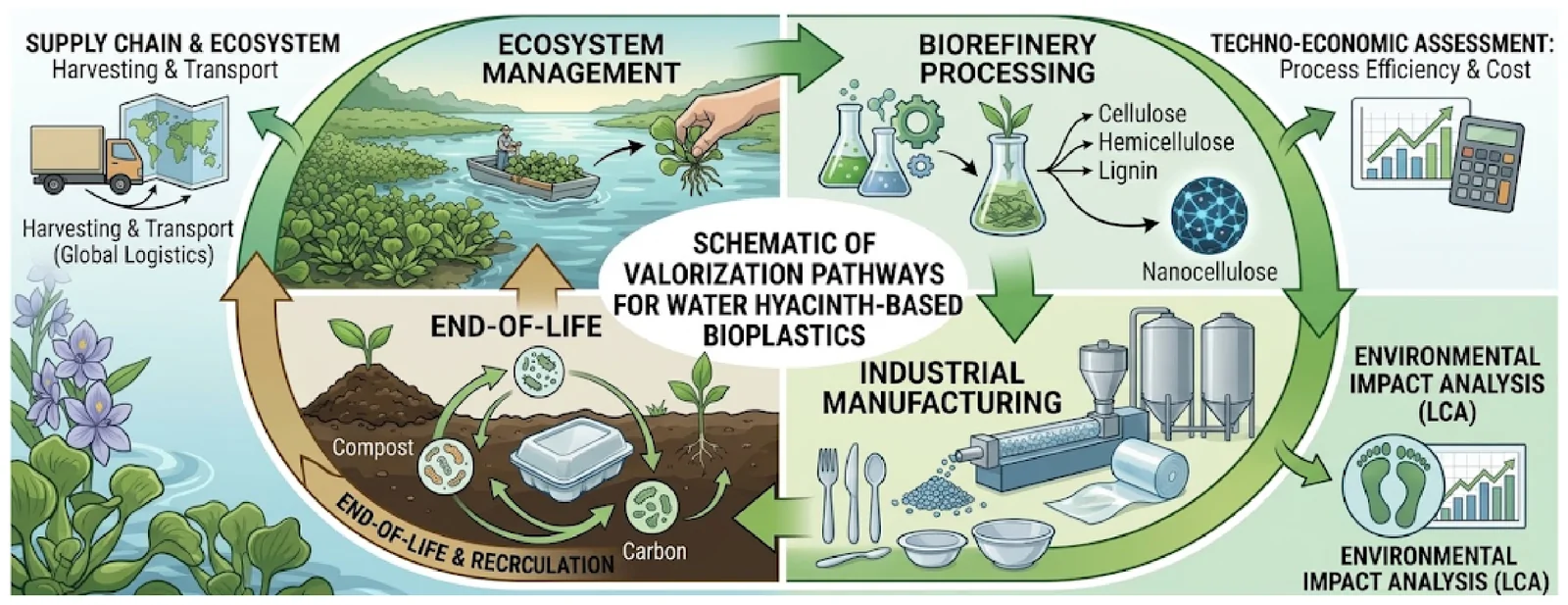
Conceptual circular-economy loop for water hyacinth bioplastics, linking ecosystem management and harvest, biorefinery processing, industrial manufacturing and end of life.

Producer prices for feedstocks will primarily determine whether they are considered economically viable. Where removal of invasive biomass is publicly funded, the effective price of the biomass to a processor may be low or negative, because the party that clears the water body may be paid to deliver it and not charged to dispose of it. This depends on local policy and contracts. Drying, transport and pretreatment would offset part of this advantage. We found no techno-economic study of water hyacinth bioplastics. Related work on water hyacinth biorefining in the Lake Tana region estimated a feedstock cost of USD 24.40 per tonne of dry matter [19], and a policy framework for Ethiopia discusses how removal, markets and regulation could be aligned [20]. Competitiveness with starch- or PLA-based products at a small scale (below about 20 tonnes per day) is therefore a hypothesis and not a finding. Figure 4 shows the circular-economy loop proposed for water hyacinth bioplastics; it is a conceptual diagram and implies no quantitative result.

## 6. Future Research Priorities

Seven principal research gaps constrain the advancement of water hyacinth (*Eichhornia crassipes*) as a viable bioplastic feedstock. First, the evidence base lacks ISO 14040/44-compliant life cycle assessments (LCAs) published in peer-reviewed outlets. Without functional unit-based comparative LCAs benchmarking water hyacinth carboxymethyl cellulose (CMC) films against conventional alternatives such as LDPE and sugarcane-derived PLA, inclusive of system-boundary sensitivity analyses for biomass removal, claims of environmental superiority remain empirically unsupported and untenable for green-labelling or procurement decisions.

Second, substantial inter-study variability in published compositional data reflects, in part, the absence of standardized biomass sampling, drying, and analysis protocols analogous to TAPPI or NREL frameworks. Until such protocols are developed and widely adopted, cellulose yield and quality data cannot be reliably compared across geographic or seasonal contexts, impeding the formation of a coherent scientific knowledge base. Reports should also state the origin of the biomass, the season of harvest and its metal content.

Third, end-of-life biodegradation data remain dominated by accelerated laboratory protocols. Field-based trials capturing degradation under realistic conditions, including UV exposure, variable soil moisture, seasonal temperature fluctuation, and natural microbial community variability, are largely absent, yet are essential for substantiating performance claims to agricultural end-users and regulatory bodies.

Fourth, food-contact safety remains uncharacterized in most published studies, reflecting the prevailing materials-science orientation of the field. For packaging applications, the migration behaviour of residual processing chemicals, including bleaching agents, alkali residues, borax crosslinkers, glycerol plasticisers, and surface modification reagents, into food simulants must be systematically quantified and benchmarked against EU regulation 10/2011 and FDA 21 CFR migration limits. This constitutes a technical prerequisite rather than an optional refinement.

Fifth, existing techno-economic analyses are scarce and not calibrated to decentralized smallholder cooperative models appropriate for tropical low-income contexts. Modelling frameworks that integrate harvest logistics, seasonal biomass availability, community-scale processing costs, and market price scenarios for target applications in sub-Saharan Africa and South-East Asia would significantly inform development finance allocation and technology transfer policy.

Sixth, prevailing biorefinery designs discard hemicellulose-rich and lignin-containing co-fractions generated during cellulose extraction. Systematic valorization of these streams, including fermentation of hemicellulose to xylose-based platform chemicals and exploitation of water hyacinth lignin as an antioxidant additive or biopolymer building block, would substantially enhance both the economic viability and environmental performance of integrated water hyacinth biorefinery systems.

Seventh, structure–property data at the molecular level are largely missing for water hyacinth materials. Studies should report the composition and crystallinity of the cellulose used and characterize the filler–matrix interaction by spectroscopic, thermal, dynamic mechanical and X-ray methods, so that the effect of the matrix can be separated from the effect of the natural variability of the filler.

## 7. Conclusions

This review has looked at water hyacinth as a source of cellulose for polymer composites and bioplastics. It is a renewable and widely available lignocellulosic resource, and the ecological and economic setting of its removal provides a reason to look for uses. The technical case is only partly made. Cellulose can be isolated from water hyacinth by alkaline, bleaching and organosolv routes, with purities from about 53% in a simple alkaline–bleaching isolate to about 93 wt% after an optimized organosolv–alkaline process, and films and composites with starch, PVA, CMC and other matrices have been produced at laboratory scale. Their reported tensile strengths are low and their elongation is far below that of LDPE, so equivalence with LDPE has not been shown, and at present the materials look suited only to limited, low-load uses. Mass loss in soil-burial tests has been reported, but mineralization and field behaviour have not been established. Suitability for food-contact, agricultural and biomedical use still has to be tested, including the control of metals and other contaminants taken up from polluted water. The circular-economy argument is plausible, because harvest is both a source of material and a form of ecosystem management, and where removal is publicly funded the feedstock may be cheap or even paid for. No techno-economic study or ISO-compliant life-cycle study has confirmed this. A decentralized production model, with cooperative harvesting and primary processing, regional cellulose hubs and urban distribution, could create livelihoods in the tropical countries where water hyacinth and plastic pollution are both severe.

The HEFD scheme used in this review is a conceptual way of organizing the evidence along the value chain. It is not a validated model, and it does not by itself show an advance over conventional biorefinery thinking. Applying it does show where the evidence is thin: we found no ISO-compliant LCA data, no standardized biomass characterization protocols, no long-term field biodegradation studies, and no food-contact safety data for realistic formulations. These gaps can be closed with targeted studies, and closing them is the necessary next step before water hyacinth materials can be judged against established plastics. The narrative of water hyacinth has long been one of destruction and costly management failure. This review suggests a different one, in which an ecological problem and a materials problem might be addressed together within a circular bioeconomy, once the evidence gaps are filled.

## Figures and Tables

**Table 1:** Reported composition of water hyacinth biomass and fiber (% of dry matter, unless stated). Values come from different plant parts, sites, and analytical methods and are not directly comparable.

Context/Region	Material Analysed	Key Composition	References
Asia (India)	Whole biomass (WHB); values quoted by the authors from an earlier study	Cellulose ~20%, hemicellulose ~33%, lignin ~10%	[27]
Asia (India)	Fibre from petiole/stem	Cellulose 62.15%, hemicellulose 14.82%; low wax 0.35%	[28]
Asia (Indonesia)	Isolated cellulose	Cellulose 53.45%, lignin 24.43%, hemicellulose 22.12% in isolated fraction (method-dependent)	[23]
Tropical Africa (Cameroon)	Extracted fibre	Cellulose ~68.3–68.4%, hemicellulose 11.3%, lignin 7.2–7.4%, pectin 4.8%, ash 7.8%	[29]
Global review	Typical WH biomass	Low lignin (<10%) and high cellulose (~20%) and hemicellulose	[30]

**Table 2:** Biodegradation measurement methods, metrics, and representative studies for water hyacinth-derived bioplastics and biomass in anaerobic digestion.

Study/Material	Biodegradation Measurement Method	Metric Type	Example Measures	References
Water hyacinth CMC–tapioca starch bioplastic films	Physical disintegration—weight loss in soil burial at 10 cm depth (gravimetric); time to visible decomposition	Weight loss (%)	% weight loss after burial; time to complete decomposition	[41]
Water hyacinth biomass—anaerobic digestion	Mineralisation—CH_4_/biogas yield in batch or semi-continuous digesters (respirometry/volumetric measurement)	Gas yield; BD (%); Kinetics; Process stability	L CH_4_ or biogas kg^−1^ VS; BD as % of the theoretical yield; lag time, max rate, R^2^; pH/VFA effects (qualitative)	[44,45]
Bioplastics/compostable polymers—controlled composting	Mineralisation—CO_2_ evolution under controlled composting conditions (ISO 14855/ASTM D5338 protocol)	BD (%); CO_2_ evolution rate	% theoretical CO_2_ released; rate curve vs. reference material (microcrystalline cellulose)	[42,46,47]

**Table 3:** Harvest–Extract–Formulate–Deploy (HEFD) Valorization Framework for Water Hyacinth Bioplastics.

HEFD Layer	Key Decision Variables	Technical Outputs	Cross-Cutting Constraints
H—Harvest	Timing (season, growth stage); harvest method (manual, mechanical); dewatering; drying protocol	Dense infestations yield large fresh biomass that varies by site and season (not quantified here). Fresh biomass is about 90–95% water, so dewatering and drying to below about 12% moisture is needed before chemical treatment (literature range and assumption).	Feedstock costs may be low or negative when removal is publicly funded (hypothesis; no techno-economic study confirms this). Logistics costs are likely to dominate diffuse infestations (expectation). Screening for pathogens and heavy metals is needed when biomass comes from polluted water (see Section 2.2).
E—Extract	Pretreatment method (alkali, acid, organosolv, enzymatic); bleaching agent; extraction temperature and duration; cellulose crystallinity target	Cellulose purity of 53.45% (alkaline–bleaching isolate) and 92.98 wt% (optimized organosolv–alkaline process), with crystallinity index rising from 37.92% to 73.52% in the latter [22,23]. Extraction yield of 19.38% for nanofibril preparation [48]. These are individual experiments, not general ranges.	Deep eutectic solvents may reduce toxicity but increase cost and require solvent recovery [49] (expectation). The reviewed sources have not quantified the energy demand and wastewater load of water hyacinth cellulose isolation.
F—Formulate	Matrix polymer (starch, PLA, chitosan); cellulose loading (wt%); plasticiser identity and concentration; crosslinking agent; processing method (casting, extrusion, compression moulding)	Tensile strength up to 2.55 MPa at 12% cellulose in banana peel starch [33]. The optima differed between the systems (4%, 6%, and 12% cellulose in the cited studies) [33,34]. Elongation and water-vapour transmission data were not consistently reported. These were individual experiments.	Interfacial adhesion is crucial, and surface treatment (alkali, silane) increases fiber–matrix compatibility; food-contact compliance requires the absence of processing chemical residues.
D—Deploy	Target application (packaging, mulch film, hydrogel, biomedical device); performance specification; end-of-life pathway (composting, soil burial, anaerobic digestion)	Mass loss of 63.18% in the best banana peel starch case [33] and near-complete disintegration within 120–144 h of burial for a CMC–starch film [41]; both are mass-loss results (disintegration), not mineralisation. Compliance with EN 13432 has not been shown for water hyacinth materials in the sources reviewed.	Different rules apply to each use: food-contact plastics (Regulation (EU) No 10/2011), compostable packaging (EN 13432), biodegradable mulch films (EN 17033), fertilizing products (Regulation (EU) 2019/1009), and medical devices (ISO 10993-1) [50,51,52,53,54]. LCA to ISO 14040/14044 is increasingly required in procurement.

Source: Authors’ synthesis of the studies cited in this review. Entries described as individual experiments come from single studies; the others are expectations or assumptions, as stated. The framework is conceptual and has not been validated.

**Table 4:** Reported composition and performance of water hyacinth-derived materials, as stated in the source studies.

Product System	Material Composition	Key Performance Data	Application Target	Reference
Water hyacinth CMC–starch film	Water hyacinth CMC blended with tapioca starch and glycerol (2–6% glycerol); soil-burial test at 10 cm depth	About 10% weight loss in 24 h; reported fully decomposed after 120–144 h depending on glycerol content (weight loss, not mineralization)	Packaging (proposed)	[41]
Starch–CNC composite	Corn starch with water hyacinth CNC at 0, 2, 4, 6, 8 and 10%	Tensile strength and elongation changed non-linearly with CNC content; greatest mass reduction at 6% CNC on day 10	Packaging (proposed)	[34]
Starch–cellulose film	Kepok banana peel starch with water hyacinth cellulose at 0, 4, 8, 12 and 16%	Highest tensile strength 2.55 MPa at 12% cellulose; greatest mass loss (63.18%) and water absorption (54.93%) at 4% cellulose	Bioplastic film	[33]
PVA/gelatin–CNC film	Polyvinyl alcohol/gelatin blend with water hyacinth CNC	Packaging and degradability properties evaluated; see source for values	Packaging films	[58]
Fibre-reinforced cutlery	Water hyacinth fibre-reinforced biodegradable material, moulded as cutlery	Proposed as a replacement for single-use plastic cutlery; see source for strength and degradation data	Single-use food service	[65]
Thermoplastic starch biocomposite	Water hyacinth-reinforced thermoplastic starch	Development and characterization reported; see source for values	Biocomposite	[66]
Cellulose nanofibril film	Cellulose nanofibrils from water hyacinth, prepared by chemical purification and mechanical fibrillation	Extraction yields 19.38%; crystallinity index up to 7.5% higher and film tensile strength up to 50% higher after mechanical processing (relative increases)	Nanocellulose films	[48]
Reference system (not water hyacinth)	Thermoplastic starch with CNC up to 50 wt%; glycerol or sorbitol plasticizer	Tensile strength 3.7 to 7.1 MPa (40% glycerol) and 11.1 to 27.1 MPa (30% sorbitol) with CNC; water vapour permeability fell up to 20 wt% CNC	Shows the effect of matrix and plasticizer	[37]

WH = water hyacinth; CNC = cellulose nanocrystals; CMC = carboxymethyl cellulose; PVA = polyvinyl alcohol; WVTR = water vapour transmission rate.

## Data Availability

The original contributions presented in this study are included in the article. Further inquiries can be directed to the corresponding author.

## Nomenclature

The following abbreviations are used in this manuscript:

ASTM	American Society for Testing and Materials
BD	biodegradability
CFR	Code of Federal Regulations
CMC	carboxymethyl cellulose
CNCs	cellulose nanocrystals
CNFs	cellulose nanofibrils
EN	European Norm
EU	European Union
FDA	Food and Drug Administration
HEFD	Harvest–Extract–Formulate–Deploy
ISO	International Organization for Standardization
LCA	life cycle assessment
LDPE	low-density polyethylene
PLA	polylactic acid
PRISMA	Preferred Reporting Items for Systematic Reviews and Meta-Analyses
PVA	polyvinyl alcohol
VFAs	volatile fatty acids
VSs	volatile solids
